# Neuroprotection unveiled: melatonin mitigates apoptotic pathways in traumatic brain injury

**DOI:** 10.3389/fneur.2025.1551449

**Published:** 2025-05-26

**Authors:** Sezer Onur Gunara, Mehmet Yigit Akgun, Ugur Seker, Ege Anil Ucar, Ozkan Ates, Ibrahim Basar

**Affiliations:** ^1^Department of Neurosurgery, Koc University Hospital, Istanbul, Türkiye; ^2^Spine Center, Koc University Hospital, Istanbul, Türkiye; ^3^Department of Histology and Embryology, Mardin Artuklu University, Mardin, Türkiye; ^4^Department of Neurosurgery, Dicle University, Diyarbakir, Türkiye

**Keywords:** traumatic brain injury, melatonin, apoptosis, neurodegeneration, mice

## Abstract

**Objective:**

This study investigated the neuroprotective effects of melatonin in mice subjected to traumatic brain injury (TBI), focusing on caspase-dependent apoptotic signaling pathways.

**Materials and methods:**

A total of 21 mice were divided into three groups: control, trauma (TBI), and trauma + melatonin (TBI + M). TBI was induced in the TBI and TBI + M groups via a free-fall impact on the frontal lobes. A single dose of 10 mg/kg of melatonin was intraperitoneally administered to the TBI + M group. Brain tissues were collected for histological evaluation and immunohistochemical analysis of apoptotic proteins.

**Results:**

The control group showed normal brain morphology, while the trauma group exhibited significant tissue loss and demyelination. The TBI + M group demonstrated reduced demyelination compared to the trauma group. An immunohistochemical analysis revealed increased expression of Bax and decreased expression of Bcl-2 in the trauma group, both of which were mitigated by melatonin treatment. The expression levels of caspase-3 and caspase-9 were elevated in the trauma group, whereas the TBI + M group showed expression levels comparable to the control group.

**Conclusion:**

TBI increased apoptotic protein expression, indicating neurodegeneration. The administration of melatonin at 10 mg/kg attenuated TBI-induced apoptosis and demyelination while promoting anti-apoptotic protein expression in the experimental model. These findings suggest a potential therapeutic role for melatonin in the management of TBI.

## Introduction

Traumatic brain injury (TBI) is a significant cause of mortality and disability, particularly among individuals aged 1–44 years, necessitating prolonged treatment and care (1). It is a leading cause of neurological sequelae among individuals under 45 years. While the primary injury at the time of trauma is the primary cause of mortality, the secondary injury occurring afterward plays a crucial role in both mortality and morbidity (2, 3).

Primary injury following trauma can manifest as a variety of clinical conditions, ranging from scalp injuries to intracranial hemorrhages, while secondary injury emerges subsequent to the primary insult and entails a complex pathophysiological process (4). The prognosis of secondary tissue damage significantly influences outcomes, and efforts to prevent or mitigate secondary damage may lead to a substantial reduction in patient mortality and morbidity (5–8).

Agents such as melatonin show promise in preventing or reducing secondary damage by inhibiting the formation of reactive oxygen species or diminishing their effects (9, 10). Given the relatively weak antioxidant mechanisms in the brain compared to other organs, post-trauma supportive therapy is of paramount importance. Prevention of oxidative damage to the brain post-injury may facilitate the recovery of brain function or minimize damage (11–13).

This study aimed to investigate the neuroprotective effects of melatonin in mice subjected to traumatic brain injury, focusing on its mechanisms involving both intrinsic and extrinsic caspase pathways.

## Materials and methods

This study was approved by the Ethics Committee for Experimental Animal of Dicle University with decision number 2020/34. The mice were obtained from the Experimental Animal Production and Research Center of Dicle University, and the experimental part of the study was carried out at the Medical Faculty Experimental Animal Production and Research Laboratory of Dicle University. All animals received humane care in compliance with the principles of laboratory animal care established by the National Academy of Sciences. All methods are reported in accordance with the ARRIVE guidelines. In addition, animal procedures were performed according to the “Guide for the Care and Use of Laboratory Animals” principles.

Ensuring animal welfare in experimental research is essential for both ethical and scientific reasons. We provided a suitable shelter that protected the animals from both physical and emotional discomfort, as well as opportunities for the animals to enjoy and engage in behaviors and activities that are considered natural for the species. Furthermore, we ensured that they experienced an emotionally healthy environment.

### Animal preparation and grouping

A total of 21 healthy adult female Wistar mice, weighing 30–50 g and not previously used in any study, were used in the experiment. The animals were acclimated to laboratory conditions for 1 week before the experiment and were maintained under a controlled environment with a 12-h light/dark cycle and with free access to food and water. The animals were randomly divided into three groups (n = 7 per group):

Control group: The mice did not undergo any trauma or treatment.

Trauma group (TBI): The mice were subjected to TBI without any subsequent treatment.

Trauma + melatonin group (TBI + M): The mice were subjected to TBI and received melatonin treatment.

### TBI induction

TBI was induced using a free-fall weight-drop model, which is widely recognized for producing a consistent and reproducible injury. The mice were anesthetized with an intraperitoneal injection of ketamine (50 mg/kg) and xylazine (10 mg/kg) to minimize pain and stress during the procedure. Once anesthetized, the mice were placed on a foam bed to absorb the impact and stabilize the body. A 30-g weight was dropped from a height of 80 cm onto the exposed skull to induce TBI. The impact site was the frontal lobe region, ensuring a consistent location of injury across all animals.

### Melatonin administration

Melatonin (Cat. no: M5250, Santa Cruz Biotechnology, DTX, USA) was administered intraperitoneally at a single dose of 10 mg/kg, 30 min post-injury. It was dissolved in 10 μl of absolute ethanol and then diluted 30 times with saline to achieve the desired lowest concentration of ethanol. The trauma + melatonin group received this treatment, while the trauma group received an equivalent volume of saline only. One hour after the single dose of melatonin, the mice were sacrificed through exsanguination, and their brains were quickly removed and processed for further analysis.

### Measurement of serum s100ß levels using ELISA

Serum s100ß levels were measured to assess brain injury, as s100ß is a specific marker for astrocytic glia cells in brain injury (14, 15). For this purpose, a commercially produced ELISA kit (Cat. no: 201-02-1102, SunRed Biotechnology, Wuhan, China) was used, and all steps were performed according to the manufacturer’s directions. At the end of the experiment, the obtained blood samples were centrifuged, and the serum was collected for ELISA. The s100ß standard was diluted according to the instructions in the datasheet and added to the wells. After loading the standard into the wells, 40 μl of the serum, 10 μl of the antibody, and 50 μl of streptavidin HRP from the ELISA kit were added to the unknown, blank, and standard wells according to the manufacturer’s instructions. The samples were incubated at 37°C for 60 min. The wells were rinsed with the wash buffer from the ELISA kit, and chromogens A and B were added to the wells. After a 10-min incubation period, a stop solution was added to all wells, and optical density (OD) was measured with a microplate reader at a wavelength of 450 nm. The obtained OD values were converted into quantitative results, taking into account the blank well result and the standard curve. The results were expressed as ng/ml.

#### Histological analysis

The collected tissue samples were processed following a routine tissue processing protocol (16). The brain tissues were fixed in 10% buffered formalin for 48 h, followed by routine processing and embedding in paraffin wax. Serial coronal sections (5 μm thick) from the frontal cortex were cut and stained with hematoxylin and eosin (H&E) and Luxol fast blue for a histological examination. H&E staining was used to evaluate the general morphology and the presence of necrosis, while Luxol fast blue staining was used to assess myelination and demyelination. Demyelination of the nerve was assessed in 21 randomly selected regions from each group, based on the criteria outlined in a previously published study. The criteria were as follows: 0 = no change in myelination, 1 = 25% demyelination, 2 = 25–50% demyelination, and 3 = 50–100% demyelination or complete loss of myelination in the white matter just above the cerebral cortex (17).

#### Immunohistochemical analysis

An immunohistochemical analysis was performed to evaluate the expression of apoptotic markers using mouse monoclonal antibodies against Bax (Catalog number: sc-7480; Santa Cruz Biotechnology, USA), Bcl-2 (Catalog number: sc-7382; Santa Cruz Biotechnology, USA), caspase-3 (Catalog number: sc-56053; Santa Cruz Biotechnology, USA), and caspase-9 (Catalog number: sc-56076; Santa Cruz Biotechnology, USA). Paraffin-embedded sections were deparaffinized, rehydrated, and subjected to antigen retrieval using a citrate buffer (pH 6.0). Endogenous peroxidase activity was blocked with hydrogen peroxide, and non-specific binding was prevented by incubating the sections with normal serum. The sections were then incubated overnight at 4°C with primary antibodies against Bax, Bcl-2, caspase-3, and caspase-9 (dilution 1:100). After washing, the sections were incubated with biotinylated secondary antibodies and then with the avidin–biotin–peroxidase complex. The chromogenic reaction was developed using diaminobenzidine (DAB), and the sections were counterstained with hematoxylin. The immunohistochemical staining was evaluated under a light microscope, and the percentage of positively stained tissue immunodensity was measured as previously described (18). For this purpose, the immunodensity analysis was performed on 21 randomly selected sections from each animal. The DAB positivity index was measured using ImageJ software, and the results were compared to the total section area. The obtained immunodensity results were converted to percentages, and the immunodensity measurements were considered statistically significant (19).

#### Statistical analysis

All statistical analyses were performed using IBM SPSS 23.0 software (IBM Corp., Armonk, NY, USA). Data were presented as mean ± deviation (SD). An ordinary one-way ANOVA test was used to analyze the differences between the groups. A *p*-value of less than 0.05 was considered statistically significant.

## Results

### Serum s100ß level results

**Figure 1:**
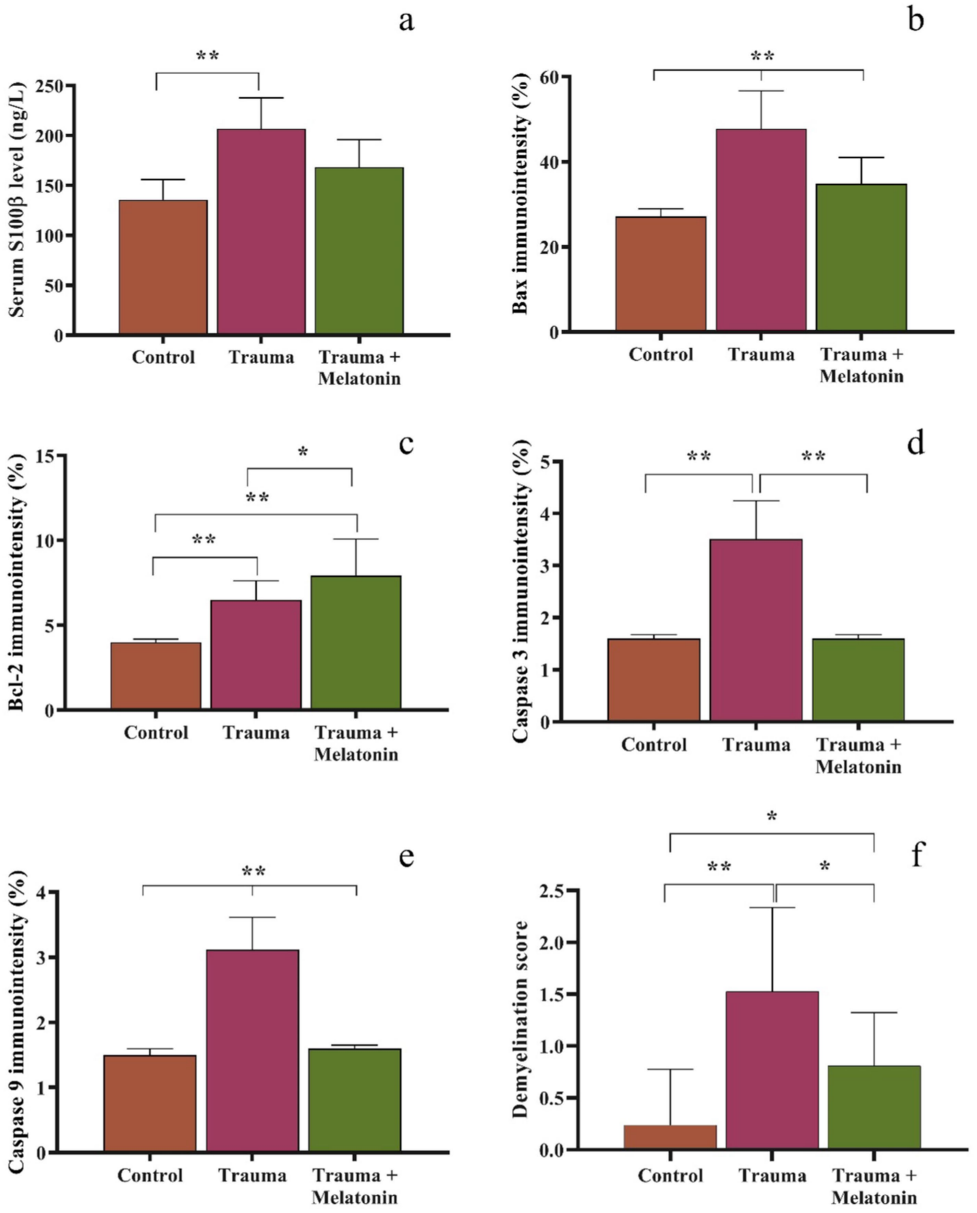
Graphical representation of serum s100ß **(a)**, tissue immunodensity analysis of Bax **(b)**, Bcl-2 **(c)**, caspase 3 **(d)**, and caspase 9 **(e)**, and demyelination scoring in the Luxol fast blue-stained brain sections **(f)**. Different symbols between or above the bars indicate statistical significance. *p < 0.05, **p < 0.01.

Regarding serum s100β levels, the concentration in the control group was 135.78 ± 20.06 ng/L. In the TBI group, serum s100β levels were upregulated to 206.83 ± 30.93 ng/L, and the results of this group were significantly (*p* = 0.001) different from those of the control group. Serum s100β levels in the TBI + M group were measured at 168.50 ± 27.44 ng/L, and our statistical analyses indicated that the results for this group were similar to both the control (*p* = 0.079) and TBI (*p* = 0.089) groups. A graphical representation of the statistical analyses is shown in Figure 1.

#### Histopathological findings

**Figure 2:**
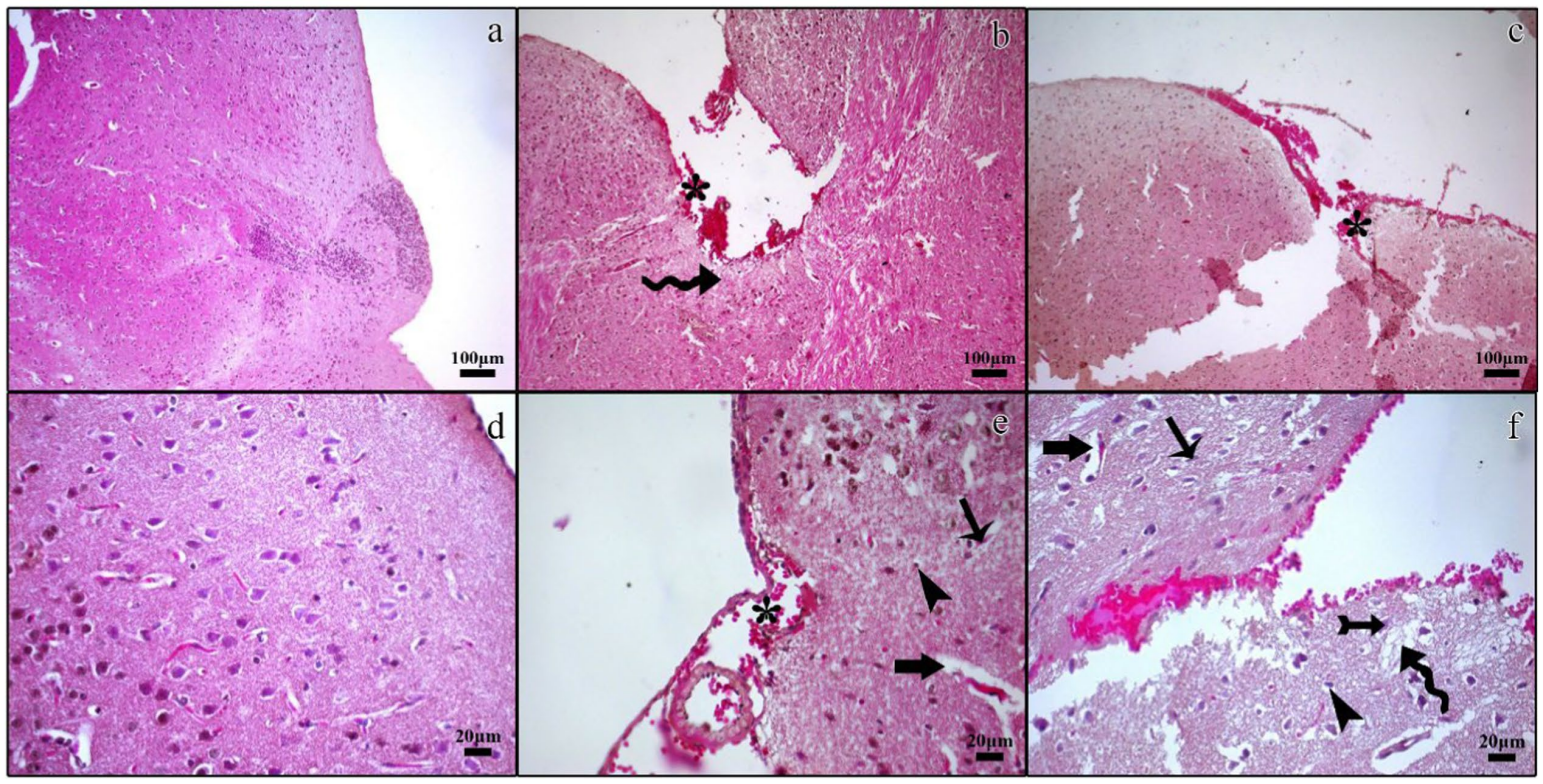
Histopathological micrographs of the control **(a,d)**, TBI **(b,e)**, and TBI + M **(c,f)** groups. Tissue loss and bleeding in the traumatic regions of the cerebral cortex and subarachnoid cavity (asterisk), edematous cerebral tissue (curved arrow), nuclear pyknosis in cerebral neurons (arrow) and glia cells (arrowhead), increased perivascular space (thick arrow), regular neuronal morphology in the traumatic region (tailed arrow). Staining: Hematoxylin and eosin. Bar: 100 μm **(a–c)** and 20 μm **(d–f)**.

**Figure 3:**
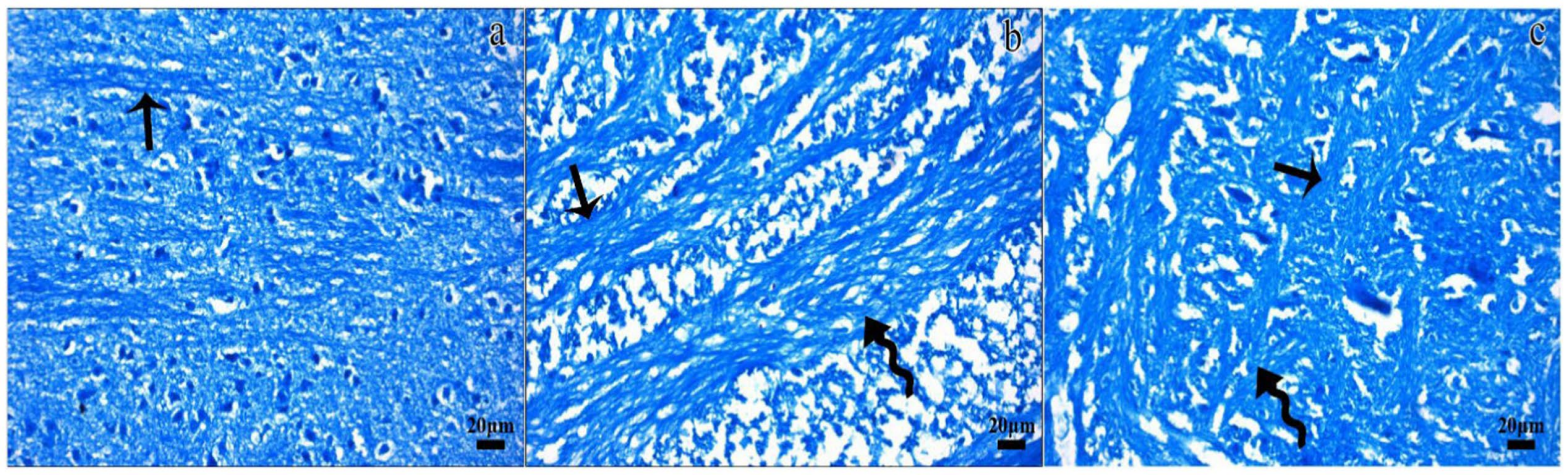
Representative micrographs of the Luxol fast blue-stained sections in the control **(a)**, TBI **(b)**, and TBI + M **(c)** groups. Dense staining (arrow) indicated normal myelinated nerve fibers in the white matter in the control group. Luxol fast blue staining for myelination was dramatically reduced in some areas of the white matter in the TBI group (curved arrow), despite the presence of regular nerve fibers. Myelinated fibers (arrow) and areas of demyelination (curved arrow) were observed in the white matter of the TBI + M group. Staining: Luxol fast blue. Bar: 20 μm.

In the control group, histological examination revealed a normal architecture of the cerebral cortex, with intact neurons, neuroglial cells, and myelinated nerve fibers. No evidence of necrosis or demyelination was observed (Figures 2a,b, 3a).

In the trauma group, there was extensive brain tissue loss and significant demyelination. The H&E staining showed widespread neuronal necrosis, gliosis, and vacuolization, indicating severe brain damage. The Luxol fast blue staining revealed a marked decrease in myelinated fibers, suggesting significant demyelination (Figures 2b,e, 3b).

A histological analysis demonstrated reduced tissue damage in the trauma + melatonin group compared to the trauma group. There was a noticeable decrease in necrotic neurons and gliosis, and the overall brain architecture was better preserved. The Luxol fast blue staining showed a higher density of myelinated fibers compared to the trauma group, indicating that melatonin treatment mitigated the extent of demyelination (Figures 2c,f, 3c). The demyelination scoring analysis indicated that the demyelination scores for the control and TBI groups were 0.24 ± 0.54 and 1.52 ± 0.81, respectively. The results between the control and TBI groups were significantly different (*p* = 0.000). The statistical analysis of demyelination scoring in the trauma + melatonin group showed a score of 0.81 ± 0.51. The results of this group were significantly different from those of the TBI (*p* = 0.002) and control (*p* = 0.014) groups.

#### Immunohistochemical analysis

##### Bax

**Figure 4:**
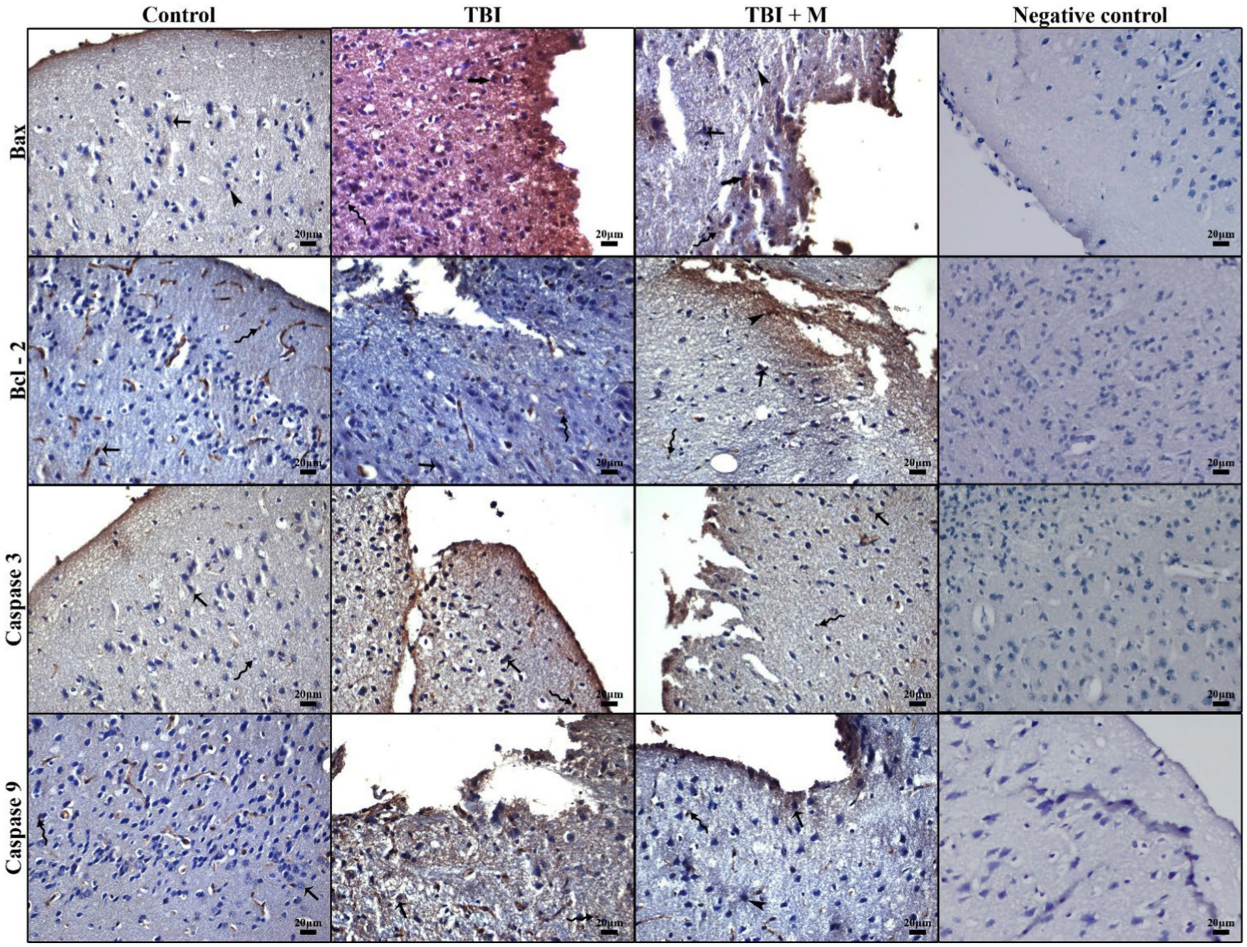
Representative micrographs of immunohistochemical staining for Bax, Bcl-2, caspase 3, caspase 9, and the negative control. Low immunopositivity in cerebral neurons (arrow), strong immunodensity in neurons (thick arrow), intense immunopositivity in glial cells (curved arrow), and low immunopositivity in neuroglia cells (arrowhead). Staining: Immunohistochemistry. Counterstaining: Hematoxylin. Bar: 20 μm.

The expression of the pro-apoptotic protein Bax was significantly increased in the trauma group (47.79 ± 8.86%) compared to the control group (27.12 ± 1.83%) (*p* = 0.000). IBax expression was significantly reduced (36.20 ± 6.20%) in the trauma + melatonin group compared to the trauma group, suggesting that melatonin treatment attenuated the apoptotic response (Figure 4).

##### Bcl-2

The expression of the anti-apoptotic protein Bcl-2 was significantly higher in both the trauma (6.50 ± 0.13%) and trauma + melatonin (7.91 ± 2.17%) groups compared to the control group (4.00 ± 0.18%) (*p* = 0.000). In addition, the results between the TBI and trauma + melatonin groups were significantly different (*p* = 0.037). These observations demonstrate that the increased Bcl-2 expression in the trauma + melatonin group indicated an enhanced anti-apoptotic response due to melatonin treatment (Figure 4).

##### Caspase-3

Caspase-3, an executioner caspase in the apoptotic pathway, showed significantly higher expression in the trauma group (3.51 ± 0.74%) compared to the control group (1.60 ± 0.08%) (*p* = 0.000). The trauma + melatonin group exhibited caspase-3 expression levels (2.10 ± 0.50%) similar to those of the control group (*p* = 1.000), indicating that melatonin reduced caspase-3-mediated apoptosis (Figure 4).

##### Caspase-9

Caspase-9, an initiator caspase in the mitochondrial apoptotic pathway, was also significantly elevated in the trauma group (3.12 ± 0.49%) compared to the control group (1.50 ± 0.09%) (*p* = 0.000). The trauma + melatonin group showed a reduction in caspase-9 expression (1.60 ± 0.06%), but the results in this group were significantly different compared to both the control (*p* = 0.001) and TBI (*p* = 0.000) groups (Figure 4).

## Discussion

Traumatic brain injury (TBI) is a critical health issue worldwide, leading to severe consequences, such as death and long-term disability, depending on the injury’s severity. In the United States alone, an estimated 2.5 million cases of TBI were reported in 2010 (20). However, the Food and Drug Administration (FDA) has not approved any specific treatment for TBI, and affected individuals often face functional deficits impacting their daily lives for years. Therefore, developing potential drug therapies to mitigate the effects of TBI is of utmost importance (21).

Among the numerous drugs and chemicals tested to control post-traumatic damage and improve patients’ quality of life, melatonin has emerged as one of the most promising agents (22). Melatonin’s low toxicity makes it a potential candidate for treating various diseases related to the central nervous system. As a chemical component naturally present in the central nervous system, melatonin easily crosses the blood–brain barrier and reaches target areas, activating intracellular cycles through receptor binding (23). Melatonin’s protective effects have been reported in several experimental animal models for conditions such as spinal cord injury, Alzheimer’s disease, sepsis-induced brain injury, stroke, and amyotrophic lateral sclerosis (ALS) (24).

Previous studies investigating melatonin’s neuroprotective effects on experimental TBI have shown promising results. Ding et al. reported that melatonin treatment significantly reduced neuronal degeneration and protected brain tissues in a TBI model created using a 200-g weight (25, 26). They also found that a 10 mg/kg dose of melatonin significantly decreased pro-apoptotic Bax expression and regulated cytochrome c levels, both of which are crucial in the mitochondrial apoptotic process. Our study supports these findings, demonstrating that a 10 mg/kg dose of melatonin effectively reduced TBI-induced neurodegeneration by modulating the expression of apoptotic proteins. Both studies suggest that melatonin may act as a neuroprotective agent against TBI-induced apoptotic neurodegeneration.

Lin et al. used a controlled cortical impact model to induce TBI in rats and investigated melatonin’s protective effects. They administered 5 mg/kg of melatonin to the rats, with one group also receiving a mitophagy inhibitor, 3-methyladenine. Their findings indicated that melatonin triggered mitophagy via the mTOR pathway, thereby balancing the release of pro-inflammatory cytokine and reducing inflammation (24). Lee et al. investigated melatonin’s neuroprotective effects in a surgical brain injury model in an animal model at varying doses (5, 15, and 150 mg/kg) (27). Their results showed that lower doses (5 and 15 mg/kg) were insufficient to reduce brain edema, but a dose of 15 mg/kg significantly decreased oxidative stress. Interestingly, the highest dose (150 mg/kg) did not show strong neuroprotective effects, indicating the need for dose standardization in melatonin treatments for TBI.

Yürüker et al. studied the neuroprotective effects of melatonin in a TBI model, using a 300-g weight dropped from a height of 2 m, after administering 5 mg/kg of melatonin 1 h post-injury (28). They reported significant reductions in intracellular reactive oxygen species, caspase-3, and caspase-9 levels in melatonin-treated animals compared to the trauma group. Although our study used a different model (mice, 30-g weight dropped from 80 cm, and 10 mg/kg melatonin), both studies demonstrated melatonin’s ability to reduce caspase-3 and caspase-9 immunoexpression, suggesting similar neuroprotective mechanisms. In addition, our study observed increased Bcl-2 expression in both the trauma and melatonin-treated groups, indicating an anti-apoptotic response. This finding aligns with the hypothesis that melatonin enhances the anti-apoptotic response to TBI due to its antioxidant potential.

Neuroglial damage and demyelination in TBI have been widely reported in previous studies. In one study, even distant oligodendrocytes were affected by TBI-induced pathology, with neuroglial degeneration persisting long after the injury. Melatonin’s protective effects in demyelinating diseases, such as multiple sclerosis, have also been documented. Our study demonstrated melatonin’s protective effects on cortical neurons and glia cells, with reduced demyelination in the Luxol fast blue-stained samples from the melatonin-treated group compared to the trauma group. These findings provide novel insights to the literature, as we could not identify any previous studies specifically evaluating melatonin’s impact on myelination or demyelination in TBI models.

While numerous animal studies have investigated melatonin’s neuroprotective effects in TBI, clinical research remains limited. Ge et al. found that 15 mg/kg of melatonin improved cognitive functions and provided neuroprotection in a rat TBI model (29). Conversely, Kemp et al. conducted a clinical study on seven male patients aged 16–65 years with a history of TBI, administering 5 mg of melatonin and 25 mg of amitriptyline (30). They reported that melatonin effectively regulated sleep onset but did not significantly improve sleep duration, quality, mood, or cognitive performance. The wide age range and the lower dose, compared to successful animal studies, could explain these limitations.

In addition to regulating apoptotic signaling pathways, previously published studies have reported the promising beneficial effects of melatonin treatment on various signaling pathways. In a previously published article, Ma et al. reported that melatonin treatment in traumatic brain injury regulated the NRF2 signaling pathway and reduced ferroptosis-associated neuronal cell death in rats (31). Similarly, Rehman et al. indicated that melatonin treatment reduced neurodegeneration, oxidative stress, and neuroinflammation by modulating the AMPK/CREB signaling pathway in male mice (32). The diversity of signaling pathways involved in the neuroprotective activity of melatonin in traumatic brain injury indicates the complex nature of traumatic brain injury pathogenesis and the potential therapeutic profile of melatonin.

Comparing our study’s results with previous research highlights the variability in melatonin dosing across studies. Our 10 mg/kg dose effectively reduced brain damage, while other studies have reported varying effects with different doses. In addition, the current literature suggests sex-related variability in response to melatonin treatment. The ideal melatonin dose for TBI treatment in animals should be standardized before clinical translation.

Although our study highlights the beneficial neuroprotective effects of melatonin through the modulation of apoptotic pathways, it is important to acknowledge that excessive inhibition of apoptosis could have adverse consequences. Apoptosis serves as a critical mechanism for eliminating severely damaged or dysfunctional cells, including those with impaired autophagy. Inhibiting apoptosis excessively might enable the survival of cells that could otherwise contribute to secondary neurodegeneration or persistent neuroinflammation. Recent studies have raised concerns that strong anti-apoptotic interventions may interfere with the clearance of dysfunctional neural cells after TBI, potentially impairing tissue remodeling and recovery. Our study did not specifically evaluate autophagic dysfunction or the long-term effects of melatonin treatment on damaged cell populations. Therefore, further research is warranted to assess the balance between neuroprotection and the necessary removal of irreversibly damaged cells when using melatonin as a therapeutic strategy in TBI.

## Limitations of this study

Our study has some limitations. Despite the histological, histochemical, and immunohistochemical evidence supporting melatonin’s neuroprotective effects in TBI, these findings may not fully translate to clinical settings.

In this study, we did not examine the effect of melatonin exposure on healthy brain structure and function. However, existing literature supports the safety of this hormone on neural function, although these observations may need further validation through more detailed studies.

Our study also has some methodological limitations. For example, this experiment was performed on female mice, and there is a need to test these results in male animals.

Furthermore, the examination of serum S100ß provides important information for the current literature; however, the effects of melatonin treatment should also be tested using laboratory examination methods such as Western blotting and PCR. Although there are some promising results regarding melatonin treatment in traumatic brain injury, more detailed studies in the future are needed, which should include behavioral analyses to assess melatonin’s broader impact and clinical relevance.

## Conclusion

This study demonstrates melatonin’s neuroprotective potential in a TBI model through its antioxidant and anti-apoptotic properties. Melatonin significantly reduced demyelination and modulated apoptotic protein expression in the trauma-induced brain. While these findings are promising, further research is needed to establish the optimal dosing and completely understand the clinical implications of melatonin treatment in TBI.

## Data Availability

The original contributions presented in the study are included in the article/supplementary material, further inquiries can be directed to the corresponding author.
